# Recent Advances on the Structure and Function of RNA Acetyltransferase Kre33/NAT10

**DOI:** 10.3390/cells8091035

**Published:** 2019-09-05

**Authors:** Sophie Sleiman, Francois Dragon

**Affiliations:** Département des Sciences Biologiques and Centre d’Excellence en Recherche sur les Maladies Orphelines-Fondation Courtois (CERMO-FC), Université du Québec à Montréal, Montréal, QC H3C 3P8, Canada

**Keywords:** ribosome biogenesis, Kre33, NAT10, post-transcriptional modifications, SSU processome, nuclear pore complex, nuclear localization signal, nucleolar localization signal, Hutchinson–Gilford progeria syndrome

## Abstract

Ribosome biogenesis is one of the most energy demanding processes in the cell. In eukaryotes, the main steps of this process occur in the nucleolus and include pre-ribosomal RNA (pre-rRNA) processing, post-transcriptional modifications, and assembly of many non-ribosomal factors and ribosomal proteins in order to form mature and functional ribosomes. In yeast and humans, the nucleolar RNA acetyltransferase Kre33/NAT10 participates in different maturation events, such as acetylation and processing of 18S rRNA, and assembly of the 40S ribosomal subunit. Here, we review the structural and functional features of Kre33/NAT10 RNA acetyltransferase, and we underscore the importance of this enzyme in ribosome biogenesis, as well as in acetylation of non-ribosomal targets. We also report on the role of human NAT10 in Hutchinson–Gilford progeria syndrome.

## 1. Introduction

Mutations in the DNA sequence of the genome can be caused by many factors, including errors in DNA replication or failure of the DNA repair system of the cell, and can lead to various changes at the protein level, such as altering their expression, folding, function and stability [1]. Mutations in proteins can also alter their specificity or selectivity of interactions with other partners (e.g., proteins, nucleic acids or small molecules) [2], and their sub-cellular localization [3]. Progeroid syndromes are rare genetic diseases caused by mutations in genes coding for DNA repair factors and DNA maintenance enzymes, or by mutations of nuclear envelope factors or their interactors. For example, Werner syndrome results from mutations in the DNA helicase WRN, required for replication [4], and Cockayne syndrome is caused by mutations in the DNA repair genes *ERCC8* or *ERCC6* [5]. The best-characterized progeroid laminopathy is Hutchinson–Gilford progeria syndrome (HGPS), which arises from a mutation in the *LMNA* gene that encodes a truncated form of lamin A named progerin [6]. More recently, the nucleolar factor NAT10 has been linked to HGPS [7,8,9]; this was a fascinating discovery because NAT10 plays a critical role in ribosome biogenesis. The making of ribosomes begins within the nucleolus, continues in the nucleoplasm and terminates in the cytoplasm [10,11]. This process involves ribosomal RNA (rRNA) transcription, processing, modification and assembly reactions that are finely tuned and lead to the formation of two large ribonucleoprotein (RNP) complexes: the small and large ribosomal subunits (40S and 60S, respectively) [12]. Ribosome biogenesis implicates more than 200 non-ribosomal factors, and a large number of these proteins are part of the small subunit (SSU) processome complex. This highly dynamic RNP of 80S–90S is essential for assembly of the 40S subunit, and consists of the U3 small nucleolar RNA (snoRNA) and about 80 ribosomal and non-ribosomal proteins [13,14].

Eukaryotic rRNAs are heavily modified. Indeed, pre-rRNAs undergo two major types of modifications, 2′-*O*-ribose methylation carried out by box C/D and pseudouridylation by box H/ACA small nucleolar ribonucleoproteins (snoRNPs) [15]. In addition, some rRNA bases are methylated by specific enzymes [16]. Remarkably, acetylation of rRNA was reported more than 40 years ago and, although the presence N4-acetylcytidine has been detected in only the small subunit rRNA of eukaryotes, the exact location of this modification and the enzyme responsible for this activity has remained elusive [17,18]. Two acetylated cytosines were recently identified in 18S rRNA; these two residues are highly conserved in eukaryotes (from fungi to mammals) and the modification is carried out by RNA acetyltransferase Kre33 (yeast) and NAT10 (humans). Kre33/NAT10 also plays a critical role in pre-18S rRNA processing events, and it was found to be important for transfer RNA (tRNA) and messenger RNA (mRNA) acetylation (discussed in Section 4) [19,20,21,22,23]. As mentioned above, human NAT10 has also been linked to laminopathies because it is the target of remodelin, a chemical compound used to treat premature aging syndromes; inactivation of NAT10 corrected nuclear defects via microtubule reorganization [7]. In this review, we describe the trafficking of cargo proteins from the cytoplasm to the nucleus and nucleolus. We also discuss the ribosome maturation process, as well as the importance of Kre33/NAT10 in many cellular events, such as ribosome biogenesis, post-transcriptional modifications and translation. Finally, we discuss the relationship between NAT10 and progeria syndrome.

## 2. Trafficking of Macromolecules between the Cytoplasm, Nucleus and Nucleolus

In eukaryotic cells, the nucleus is a dynamic spatiotemporal structure that provides a critical connection between genome organization and expression (reviewed in Ref. [24]), and allows for the nucleocytoplasmic transport of many ions, small proteins and macromolecules. This process is controlled by the nuclear pore complex (NPC), a large protein complex of about 125 MDa in vertebrates and 66 MDa in yeast [25,26]. In mammalian cells, mitotic events represent an open biological process in which the nuclear envelope (NE) disassembles at the beginning of mitosis and reassembles when chromosomes segregate [27]. The nucleolus, a prominent nuclear structure responsible for ribosome production, is disorganized in prophase and reassembles at the end of mitosis [28]. In yeast cells, mitosis is considered a closed event, wherein the NE remains intact during division. In budding yeast, a small daughter cell grows from a large mother cell and conveys its own transcription program; the nucleus is round during interphase and adopts an hourglass shape during mitosis [29,30].

### 2.1. The Nuclear Pore Complex of Eukaryotic Cells

The exchange of molecules between the cytoplasm and the nucleus is dependent on the NPC, a huge protein complex that controls the import and export of macromolecules by generating a fusion between the inner and outer nuclear membrane. Cryo-electron tomography and electron microscopy show that the NPC core has a diameter of about 130 nm and a height of about 80 nm. The core appears as three porous ring densities named cytoplasmic, inner, and nucleoplasmic, according to their location [31]. The nucleoplasmic side is attached to eight extended filaments connected to a distal ring to form the nuclear basket. On the cytoplasmic side, eight flexible extensions emerge [32]. Proteomic analyses have shown that the NPC comprises about 30 different nucleoporins (or Nups), which are organized in stable sub-complexes [33]. The Y-shaped complex is the best-characterized NPC sub-complex; it comprises the majority of nuclear and cytoplasmic ring structures, and depletion of its components can abolish the formation of the NPC. The transmembrane Nups complex is embedded in the nuclear envelope. The human hsNup96 complex (or yeast Nip96 sub-complex) is located in the inner ring and is formed by five to seven Nups (reviewed in Ref. [25]). These three sub-complexes give shape to the NPC; however, the selectivity or transport barrier is mainly accomplished by about 10 specific nucleoporins that contain fiber-like extensions with phenylalanine and glycine repeats (FG-Nups or FG-repeats) [32]. FG-repeats control the nucleo-cytoplasmic exchange and only allow the diffusion of small molecules (up to 40 kDa). The deletion of FG-repeats in yeast is lethal [34]. In contrast, the transport of large cargos do not require these FG-repeats and is instead achieved by the nuclear transport receptor that recognizes a nuclear localization signal (NLS) [35].

### 2.2. Import of Cargo Proteins into the Nucleus

First identified in simian virus 40 (SV40) as a short stretch of specific amino acids, the NLS motif has been further characterized and divided into a mono-partite basic type and a bi-partite basic type, which contain mainly basic amino acids such as lysine and arginine [36]. The import of nuclear proteins through nuclear pores is mediated by an NLS and requires two essential steps: targeting to the pores and translocation. In mammals, the targeting step involves formation of a stable complex termed the nuclear pore targeting complex (PTAC), containing PTAC58 and PTAC97, also called NLS-receptors (NRs). For the translocation step, two soluble factors are required, specifically a small guanosine triphosphate hydroxylase (GTPase) called Ran and its interacting partner p10/NTF2, along with the NPC components [37,38]. NRs are formed by two subunits, NRα and NRβ, also called importins. Importin α acts as an adaptor protein and binds to the NLS, while importin β mediates the interactions with the pore complex [39]. A key regulator of nucleocytoplasmic transport is the small GTPase Ran, which belongs to the Ras-like GTPase family [39]. Ran exists in two conformational states, a GTP-bound form in the nucleus and a guanosine diphosphate (GDP)-bound state in the cytoplasm. The different conformational changes are due to localization of Ran regulatory proteins present in the two different cellular compartments, thus creating a Ran gradient across the nuclear envelope, and providing specific directionality between the nucleus and cytoplasm [32,35,40]. When Ran-GTP relocalizes to the cytoplasm, the GTPase activity of Ran is stimulated by the cytoplasmic GTPase activating protein RANGAP1, and this allows a new round of nuclear import by Ran-GPD. In the nucleus, the guanine nucleotide exchange factor regulator of chromosome condensation 1 (RCC1) is responsible for Ran-GTP production [41,42]. In addition to its critical role in the nuclear export of pre-ribosomal particles, tRNAs, microRNAs and snRNAs [43], Ran-GTP functions in several nuclear events, such as maintenance of nuclear structure and spindle assembly [42]. Aside from their role in nuclear localization, it has been reported that NLSs of ribosomal proteins (r-proteins) reside within highly diverged rRNA-binding domains and make direct contact with rRNA. These NLSs coordinate delivery of ribosomal proteins to the nascent rRNA, facilitate the binding between r-proteins and rRNA, and contribute to rRNA folding during ribosome biogenesis [44].

### 2.3. Nucleolar Localization Signals (NoLSs)

For many years, protein localization to the nucleolus was poorly understood and was believed to result from interactions with nucleolar components, such as ribosomal DNA (rDNA), rRNAs and proteins, as if nucleolar localization was more a retention process rather than genuine targeting [45]. Analyses of the nucleolar proteome led to the identification of about 4500 proteins located in human nucleoli [46], and several mRNAs and microRNAs were also detected in the nucleolus [47]. Nucleolar localization signals (NoLSs) have been identified: these sequences are rich in basic residues (at least five basic amino acids, mostly lysines and arginines), they often overlap with an NLS motif, and they can be found in bi-partite forms [48]. Martin et al. [49] discovered key differences between NLSs and NoLSs by looking at the targeting and accumulation of peptides and proteins in the nucleolus. Their results suggested that peptides containing only six positively charged arginines with a pI above 12.6 were sufficient for nucleolar localization. Several proteins were reported to have two NLS motifs; one allowing nuclear localization and the other to target the protein to the nucleolus. For example, protein phosphatase-1 inhibitor-3 was shown to be localized to the nucleoli and centrosomes in interphase HEK-293 cells. The protein has two basic stretches, one in the N-terminal region and the other in the C-terminal part. When the N-terminal NLS motif was mutated, the protein accumulated in the cytoplasm; in contrast, mutating the C-terminal “NLS” motif prevented nucleolar targeting and the protein accumulated in the nucleoplasm, suggesting it is an NoLS [50].

## 3. The Nucleolus and Ribosome Biogenesis

### 3.1. The Sub-Compartments of the Nucleolus

The nucleolus, a non-membrane and condensed substructure of the nucleus, is organized around chromosomal regions that contain rDNA. This nuclear compartment is the key site of rRNA transcription and processing, as well as ribosome assembly, but it also plays critical roles in cell-cycle progression, proliferation, cellular stress response, development and aging [51,52,53,54]. The internal structure of the nucleolus has been studied in detail by electron microscopy (EM) and three main sub-compartments have been identified. These include pale structures formed by fine fibrils, representing the fibrillar center (FC), which is surrounded by the dense fibrillar component (DFC). The FC and DFC are embedded in the granular component (GC) [55]. These different substructures are related to different steps of ribosome biogenesis. The FC contains unengaged RNA polymerase I. Nascent RNA transcripts appear at the junction between the FC and DFC. The nucleolar proteins that participate in the early stages of rRNA processing localize to the DFC, whereas proteins involved in the late stages of processing are observed in the GC [56]. In fixed yeast cells, the nucleolus occupies up to one-third of the total nuclear volume and forms a crescent-shaped zone juxtaposed to the nuclear envelope [57]. In addition, it has been reported that tri-partite organization is not general in all organisms, such as *Drosophila* and insects that lack the FC compartment. This suggests that the differences in organization between bi-partite and tri-partite nucleoli could be linked to the evolution of rDNAs, in particular to the size of the intergenic sequences [58].

### 3.2. Ribosome Biogenesis in Eukaryotes

The ribosome is a cellular nanomachine that links mRNA to tRNAs in order to synthesize proteins. Eukaryotic ribosome biogenesis is a very complex, dynamic and highly coordinated process that requires not only rRNAs and r-proteins, but also more than 200 assembly factors (AFs) and many snoRNAs [14,59]. The construction of a ribosome starts with the transcription of a long precursor rRNA (pre-rRNA) in the nucleolus by RNA polymerase I (RNA Pol I). The 5S rRNA is synthesized by RNA Pol III. In yeast, 35S pre-rRNA encodes 18S, 5.8S and 25S rRNAs, which are separated by two internal transcribed spacers, ITS1 and ITS2, and flanked by 5′ and 3′ external transcribed spacers, 5′ETS and 3′ETS.

**Figure 1:**
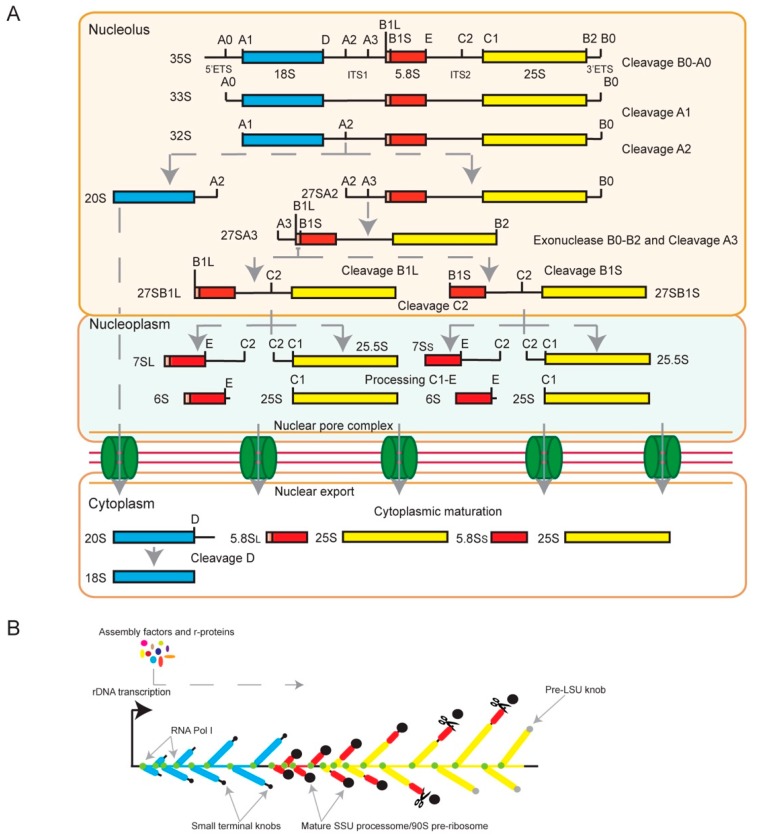
Processing of pre-ribosomal RNA (pre-rRNA) and small subunit (SSU) processome formation in the yeast *Saccharomyces cerevisiae*. (**A**) Scheme of 35S pre-rRNA processing steps. The 35S precursor is cleaved and trimmed at different sites to form the mature 18S (blue), 5.8S_L_, 5.8S_S_ (red) and 25S (yellow) rRNAs. Processing takes place in different cellular compartments (nucleolus, nucleoplasm and cytoplasm), represented by rectangles. Cleavage sites are indicated on the transcripts and are detailed in the text. The final maturation steps occur in the cytoplasm once the late precursors have been exported via the nuclear pore complex (NPC, colored in green). (**B**) Representation of SSU processome formation. The ribosomal DNA (rDNA) is transcribed by RNA polymerase I (Pol I) (green circles). When visualized by electron microscopy, actively transcribed rDNA units appear as Christmas trees, wherein chromatin forms the trunk, nascent rRNA transcripts are the branches, and the terminal knob seen at the 5′ end of transcripts is the forming SSU processome. The small black circles represent the newly formed SSU knobs and the larger black circles are the mature SSU knobs, where the 5′ETS and the 18S rRNA are packed. The large SSU knob is then cleaved at site A2 (represented by the black scissors). The large subunit precursor (pre-LSU) knobs are shown in grey circles. This schematic representation is adapted from Osheim et al. [70].

The removal of pre-rRNA spacers is a multi-step process that begins with cleavages in the 5′ETS and ITS1 at sites A0, A1 and A2 (Figure 1A). The 5′ETS is removed by cleavages at sites A0 and A1 to generate 33S pre-rRNA and 32S pre-rRNA, respectively. The cleavage at site A2 in ITS1 generates 20S and 27SA2 pre-rRNAs, thus separating pre-40S and pre-60S subunits. Mature 18S rRNA is then processed from 20S pre-rRNA in the cytoplasm by cleavage at site D, removing the D-A2 fragment. Two alternative pathways mature 27SA2 pre-rRNA, leading to the formation of 5.8S and 25S rRNAs. In the major pathway, 27SA2 is cleaved at site A3 in ITS1 by RNase mitochondrial RNA processing (MRP), forming the 27SA3 precursor. This pre-rRNA is trimmed up to site B1_S_ to form a 27SB_S_ precursor, which contains the mature 5′ end of the short form of 5.8S rRNA (5.8S_S_). In the minor pathway, the 27SA2 precursor is directly cleaved at site B1_L_ to produce the 27SB1_L_ precursor, which contains the mature 5′ end of the long form of 5.8S rRNA (5.8S_L_). The major and minor pathways undergo the same processing event at site C2 in ITS2, resulting in the formation of 7S and 25.5S precursors. The 5′ end of the 25.5S precursor is trimmed by Rat1 to produce mature 25S rRNA, and the 3′ end of the 7S pre-rRNA is processed in several steps to form mature 5.8S_L_ and 5.8S_S_ rRNAs [14,60,61] (Figure 1A).

As 35S pre-rRNA synthesis occurs, assembly factors and r-proteins bind to nascent pre-rRNAs in a hierarchical manner. Six dedicated chaperones (Tsr2, Yar1, Acl4, Rrb1, Sqt1 and Syo1) have been characterized in yeast cells and shown to have an important role in mediating a safe transfer of r-proteins into pre-ribosomal particles, as well as holding them in place during particle assembly [62,63,64]. Pre-40S and pre-60S initiate their assembly as nucleolar particles containing many factors and undergo several changes by releasing some of these factors to form mature subunits [65,66]. Pre-40S is formed by a large macromolecular complex named the SSU processome or 90S pre-ribosome [13,67]. The SSU processome is composed of more than 70 non-ribosomal proteins, including many factors with WD40 repeats used for protein–protein interactions, as well as helical repeat structures used for stabilization of RNA and proteins [14,68]. In addition, the SSU processome possibly contains four snoRNAs (U3, U14, snR30, and snR10) [69]. Disruption of diverse SSU processome components results in cleavage defects at sites A0, A1 and A2 of the pre-rRNA. Depletion experiments have revealed that a large terminal knob seen at the 5′ end of nascent transcripts in Miller chromatin spreads represents the SSU processome [13,70,71]. These knobs decorate pre-rRNAs to form the legendary Christmas trees [13,70,71] (Figure 1B).

In recent years, cryo-electron microscopy (cryo-EM) has been extensively used to study the assembly of ribosomes in *Saccharomyces cerevisiae*, the best-studied organism in the field of ribosome biogenesis [68,72,73,74,75]. Studies using pre-rRNA mimics tagged with RNA aptamers have revealed the multiple steps leading to SSU processome formation [76,77]. Assembly of the SSU processome starts with the binding of the seven-protein UtpA complex to the 5′ETS of pre-rRNA and the recruitment of the six-protein UtpB complex [78,79]. Following this binding, the U3 snoRNP (constituted of the U3 snoRNA and proteins Nop1, Nop56, Nop58, Rrp9, and Snu13) binds the nascent pre-rRNA [80]. The U3 snoRNA base-pairs with the 18S rRNA to form short duplexes [81,82]. The Mpp10 and UtpC complexes then join the preformed particle [66,83]. The 18S rRNA folds into four domains, named 5′, central, 3′ major and 3′ minor [84], and these 5′ETS components spatially separate the 18S rRNA domains to facilitate the incorporation of other enzymes and assembly factors into the SSU processome/90S particle [64]. The endonuclease Utp24 is later recruited near site A1 and directs cleavage at sites A1 and A2 [85]. Proteins such as Bud22, the dimer Bfr2/Enp2 [86], Lcp5 and Dbp4 are recruited to the 5′ domain of 18S rRNA. The U14 snoRNP also joins the 5′ domain, and acts both as a processing and modification RNP. RNA helicase Dbp4 serves as a release factor and disrupts the interaction between U14 snoRNA and 18S rRNA [87]. Then, the snR30 snoRNP is added to the central domain of 18S rRNA [66], and Emg1 methyltransferase and Nop6 assemble with the 3′ major domain [76]. Following transcription of the 3′ minor domain, the SSU processome undergoes conformational changes that trigger the release of factors binding the 5′ and the central domains, and 11 proteins (Kre33, Bms1/Rcl1, Nop14/Noc4, Utp14, Utp20, Enp1, Dim2, Rrp12 and Rrt14) enter the SSU processome [68]. Assembly finishes when the Faf1 protein joins the beginning of the ITS1 region [77]. Further elongation of the ITS1 sequence does not recruit other assembly factors to the SSU processome, indicating that only a few nucleotides of ITS1 are required for 90S pre-ribosome formation [77].

The 60S subunit is formed by 25S, 5.8S, 5S and about 46 r-proteins [88]. The 25S and 5.8S rRNAs base-pair together to form a structure composed of six conserved domains (I–VI). The pre-60S matures as it transits from the nucleolus to the nucleoplasm and cytoplasm. During this journey, about 90 AFs assemble in a dynamic and hierarchical manner [64], while ITS1 and ITS2 undergo processing. Recently, the cryo-EM structure of the nucleolar pre-60S ribosomal subunit was solved by Sanghai et al. [89] and Zhou et al. [74]. These studies illustrated the extreme complexity of 60S particle assembly; to learn more about this topic, the reader is invited to consult an excellent review by Klinge and Woolford [66].

## 4. A Variety of Targets for Kre33/NAT10

### 4.1. Formation of N4-Acetylcytidine

More than 112 different types of RNA modifications have been identified in cellular RNAs and some viral RNAs [16]. tRNAs are the most extensively modified RNAs, with 25 different types of modification identified in yeast [90]. Maturation and processing of tRNAs is essential for efficient translation. Indeed, tRNAs are transcribed as pre-tRNAs in the nucleus by RNA Pol III and undergo post-transcriptional modifications to generate mature tRNAs that deliver amino acids to cytoplasmic ribosomes. Post-transcriptional steps include processing at both the 5′ and 3′ extremities, addition of the 3′ CCA end, removal of introns (for intron-containing tRNA genes) and nucleoside modifications [91,92]. Post-transcriptional modifications are required not only for maturation, but also contribute to tRNA stability, tRNA function and the fidelity of protein synthesis when modifications are embedded in the anticodon loop region, thus maintaining a correct reading frame [93]. For example, in *S. cerevisiae*, the tRNA carboxymethyluridine(34)-5-*O*-methyltransferase Trm9 mediates methylation at the wobble position of tRNA^Arg^ and tRNA^Glu^ to enhance translation [94]. In *Escherichia coli*, the cytidine acetyltransferase TmcA acetylates tRNA^Met^ at the wobble position to ensure precise decoding of the non-initiator AUG codon [95]. tRNA^Ser^ and tRNA^Leu^ of *S. cerevisiae* have been shown to be acetylated at position 12 (ac^4^C12), a modification that requires the RNA-binding protein Tan1, which is characterized by a C-terminal THUMP domain. Both the presence of Tan1 and the ac^4^C12 modification are important for tRNA stability [96].

Most modifications in rRNAs are guided by snoRNAs and few by stand-alone enzymes; these modifications stabilize the structure of rRNAs and contribute to efficient protein synthesis [16]. The most prevalent modifications in rRNA are 2′-*O*-ribose methylation and pseudouridylation (the isomerisation of a uridine into pseudouridine, Ψ), these modifications being respectively catalyzed by box C/D and box H/ACA snoRNPs. In yeast rRNAs, 48% of modified nucleotides are 2′-*O*-Me and 40% are Ψs [97]. In addition, four SSU and six large subunit (LSU) rRNA bases are methylated in yeast [16]. Furthermore, eukaryotic 18S rRNA is unique in that it contains two acetylated cytosines. Although the presence of N4-acetylcytidine in 18S rRNA of various eukaryotes was first reported in the late 1970′s [17], their precise location remained unclear until it was discovered that the conserved GCN5-related acetyltransferase Kre33 was responsible for 18S rRNA acetylation. A number of studies have determined the critical role of yeast Kre33 and human NAT10 in acetylation of residues C1280/C1337 in helix 34 and C1773/C1842 in helix 45, as well as pre-18S rRNA processing and assembly of the 40S subunit [19,20,21,22,98]. However, Sharma et al. further demonstrated that Kre33/NAT10 carries out the ac^4^C12 modification in tRNA^Ser^ and tRNA^Leu^, and that tRNA acetylation required the intervention of the conserved adaptor protein Tan1 (THUMPD1 in humans) [22]. This also suggested a crosstalk between the modification processes of rRNAs and tRNAs [16] (see concluding remarks). Moreover, a novel role for box C/D snoRNAs snR4 and snR45 was revealed. Specifically, these snoRNAs were found to guide the enzyme Kre33 to the proper sites of acetylation in 18S rRNA, and the base pairing between the snoRNAs and 18S rRNA required the helicase activity of Kre33 [99].

**Figure 2:**
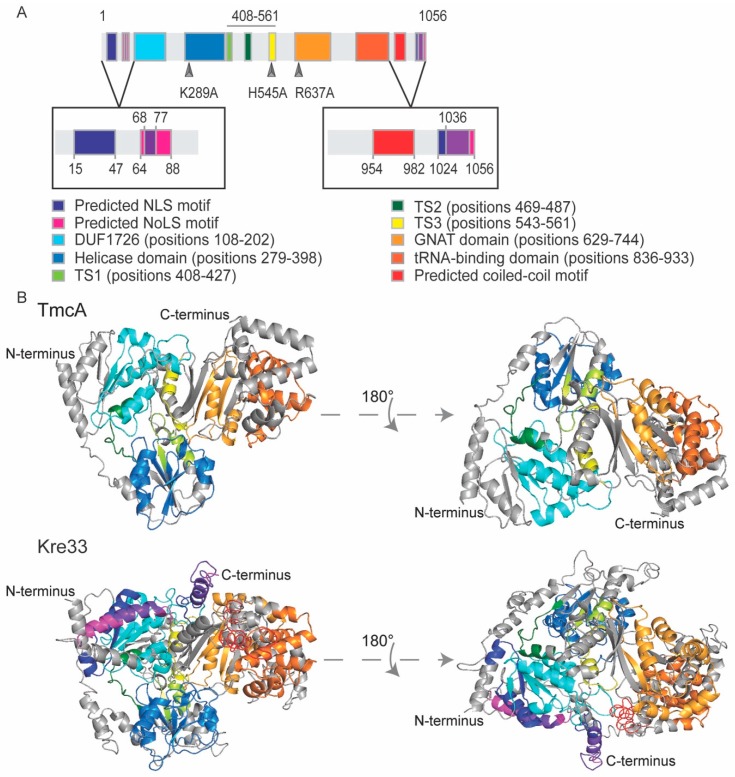
Conserved domains and motifs of Kre33. (**A**) Representation of yeast Kre33 highlighting the conserved domains/motifs. From left to right, the N-terminal extension (NTE), containing putative nuclear localization signal (NLS) and nucleolar localization signal (NoLS) motifs, colored in dark blue and magenta, respectively. Overlapping NLS and NoLS regions are shown in purple. The NTE is followed by a domain of unknown function called DUF1726 (light blue), a helicase domain (blue), three TmcA-specific motifs (TS1, TS2 and TS3) colored respectively in light green, dark green and yellow, a *N*-acetyltransferase domain (orange) and a possible tRNA-binding domain (paprika). These highly conserved domains precede a C-terminal extension (CTE) harboring a putative coiled-coil motif (red) followed by the NLS (blue) and NoLS (magenta), with overlapping regions in purple. Point mutations generated by Sharma et al. [22] are indicated. (**B**) Tridimensional representation of bacterial TmcA and yeast Kre33. Structures were modeled with I-TASSER [101]. Conserved motifs were colored using PyMOL with the color code used in (A).

**Figure 3:**
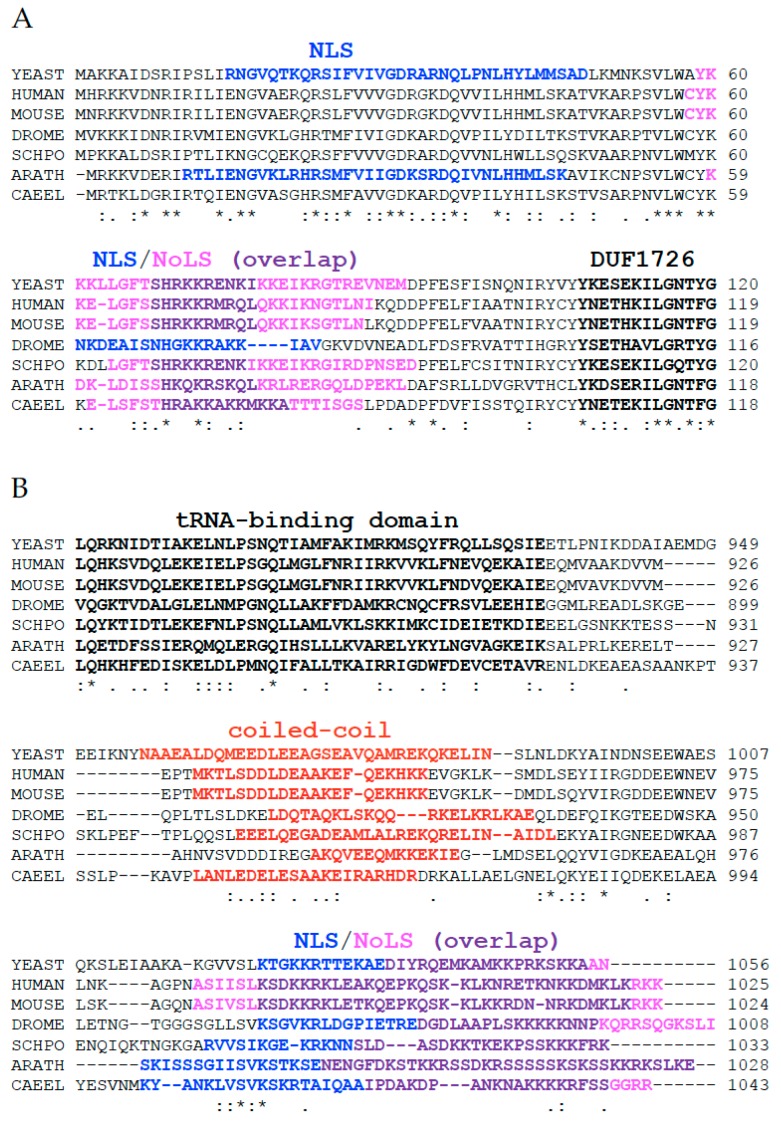
Alignment of Kre33/NAT10 from different species. Sequences from yeast, human, mouse, *D. melanogaster* (DROME), *S. pombe* (SCHPO), *A. thaliana* (ARATH) and *C. elegans* (CAEEL) were used to generate an alignment with Clustal Omega [102]. (**A**) The N-terminal extension precedes the DUF1726 (black bold letters) and harbors eukaryote-specific motifs, such as the NLS (blue) and the NoLS (magenta); overlapping NLS/NoLS sequences are in purple. (**B**) The C-terminal extension follows the tRNA-binding domain (black bold letters): it contains a coiled-coil motif (red) and finishes with the NLS (blue)/NoLS (magenta); the NLS/NoLS overlap is in purple. The putative NLS, NoLS and coiled-coil motif were identified with cNLS mapper [103], NoD [104] and COILS [105].

Kre33/NAT10 is conserved from bacteria (named TmcA) to humans. It contains multiple domains or motifs that are present in all TmcA homologs, starting with a domain of unknown function named DUF1726 near the N-terminus, followed by a helicase domain containing characteristic Walker A and Walker B motifs, three TmcA-specific motifs (TS1, TS2 and TS3), a *N*-acetyltransferase (AT) domain and a possible tRNA-binding domain. In eukaryotes, the enzyme contains additional residues at both extremities (Figure 2). Our bioinformatics searches revealed that extensions at the N-terminus (NTE) and C-terminus (CTE) of Kre33/NAT10 encompass putative motifs that are found only in eukaryotic paralogs. The NTE contains at least one putative NLS and (generally) one possible NoLS motif, and these motifs can partially overlap (Figure 3A). The CTE harbors a coiled-coil motif responsible for protein–protein interaction, followed by an NLS and an NoLS that overlap (Figure 3B).

Mutation analyses were conducted by different groups to characterize the conserved domains of Kre33/NAT10. Point mutation G285D in the Walker A motif of *Nat10* generated a temperature-sensitive phenotype in *S. pombe* and abolished acetylation of 18S rRNA [20]. In another study, a point mutation in Kre33 Walker A (K289A) reduced the growth of yeast cells, decreased acetylation activity and affected pre-rRNA processing; however, mutations in the TS3 motif (H545A) or the acetyltransferase domain (R637A) (Figure 2A) did not affect growth, but completely inhibited the acetylation of 18S rRNA and tRNAs [22]. In addition, deletion of snR4 or snR45 decreased the level of 18S acetylation, which is compatible with their role in guiding ac^4^C modification in 18S rRNA. These results demonstrate that acetyltransferase activity is not essential for growth [99]. It was shown recently that localization of NAT10 in different sub-cellular compartments is dependent on its NLS sequences. Deletion of about 30 conserved residues in the CTE of NAT10 impaired nuclear localization, leading to accumulation of NAT10 in the cytoplasm. However, deletion of the N-terminal extension of NAT10 did not affect localization, and NAT10 was restrained to the nucleolus [100]. Similar results have been observed with Kre33 in yeast cells (our unpublished data).

### 4.2. Diverse Functions of NAT10 in Human Cells

NAT10 is a member of the GNAT family of histone acetyltransferases [106,107]. It has also been identified as a telomerase activity regulator and been shown to increase the expression of human telomerase reverse transcriptase [108]. Moreover, NAT10 has been found to participate in the DNA damage response: when cells are treated with the genotoxic agents H_2_O_2_ or cisplatin, *NAT10* transcription activity and mRNA levels increase, as well as accumulation of NAT10 in the nucleus. As accumulation of NAT10 enhances cellular resistance to genotoxicity, it may be concluded that NAT10 plays a role in the response to DNA damage [109]. *NAT10* mRNA levels have also been found to be elevated in hepatocellular carcinoma (HCC) in comparison with non-cancerous tissues. In HCC, NAT10 accumulates in the nucleoplasm and cytoplasm. In contrast, NAT10 localizes to the nucleolus of normal tissues. This mislocalization suggests a relationship between NAT10 and carcinogenesis [110]. In fact, increased NAT10 expression in HCC is correlated with poor survival of patients [111].

NAT10 has been found to localize to the nucleolus during interphase and in the mitotic midbody during telophase. Depletion of NAT10 creates changes in nucleolar assembly and morphology, affecting cytokines and restraining cells to the G2/M phase [112]. In vivo acetylation and in vitro autoacetylation of NAT10 was established by Cai et al. [113]. In their study, the lysine residue at position 426 (K426) was shown to be acetylated by NAT10, and the K426R mutant failed to activate rRNA transcription, suggesting an important role of autoacetylated NAT10 in controlling rRNA transcription. NAT10 has also been found to be deacetylated in vitro and in cellulo under glucose starvation. To maintain cell survival, the cellular energy sensor AMPK activates Sirt1, which then deacetylates its downstream substrates. Sirt1 interacts with NAT10 and promotes NAT10 deacetylation. NAT10, lacking its acetyl group, thereby inhibits rRNA biogenesis, decreases energy consumption and enhances cell survival [114].

More recently, Arango et al. (2018) demonstrated cytidine acetylation in mRNAs and proposed that NAT10 was the responsible acetyltransferase. Indeed, once NAT10 was depleted, acetylation of mRNAs was abolished. Using antibodies against ac^4^C, they showed a loss of mRNA acetylation in cells knocked-down for NAT10. These cells were rescued by over-expression of the enzyme. RNA immunoprecipitation assays with ac^4^C antibodies followed by next generation sequencing revealed the distribution of ac^4^C peaks in mRNAs, showing a major peak in the 5′ untranslated region (UTR) and beginning of the coding sequence. Fewer acetylated residues were found in the rest of the coding sequence, and even less in the 3′UTR. Their results suggested that ac^4^C may play a critical role in increasing the translation level by promoting the stability of mRNAs during translation [23,115,116].

### 4.3. Laminopathies, the Hutchinson–Gilford Progeria Syndrome and NAT10

Nuclear lamins, also known as Class V intermediate filaments (IFs), are fibrous proteins that play important roles in shaping the nucleus and providing essential structural and functional properties for DNA replication, chromatin organization and cell cycle dynamics. Lamins are grouped into A-type (lamins A and C) and B-type (lamins B1 and B2) (reviewed in Ref. [117]). Lamin A is the major component of the nuclear lamina; it provides mechanical support to the nuclear envelope and is closely associated with the NPC. It has a typical IF structure, consisting of a segmented coiled-coil α-helical rod domain flanked by a short N-terminal head and a long C-terminal extension; the latter contains an NLS motif followed by an immunoglobulin fold [118]. Maturation of lamin A follows a multi-step pathway, which begins with the modification of pre-lamin A harboring a CAAX-box at its C-terminus. The cysteine of this box triggers three enzymatic reactions leading to farnesylation and carboxymethylation. The farnesylated pre-lamin A is then cleaved by ZMPSTE24, and 15 to 18 amino acids are removed from the C-terminus. The mature lamin A is then incorporated into the nuclear lamins [119,120,121,122,123].

Various diseases are associated with mutations in the *LMNA* gene, and are referred to as “laminopathies”. The most devastating of those diseases is Hutchinson–Gilford progeria syndrome (HGPS), a very rare disease that affects about one in every four million births worldwide and causes rapid aging [124]. Children with the disease appear to be normal at birth, but they die from cardiovascular diseases within approximately 13 years [125]. During their short life, they present symptoms that resemble normal aging, such as thin skin, hair loss, arthrosclerosis, cardiomyopathy, muscular dystrophy, lipodystrophy and neuropathy [126]. HGPS is caused by a single nucleotide mutation, C1824T, in exon 11 of the *LMNA* gene. In both the wild type and mutant, the nucleotide triplet codes for a glycine at position 608; however, the mutated form activates a cryptic donor splice site in the *LMNA* transcript. This new splicing site forms a truncated transcript encoding a protein that is 50 amino acids shorter in the C-terminal region, and is referred to as progerin or LAΔ50 [127]. At first, Giovannali et al. (2003) revealed by RT-PCR and western blotting experiments that only the mutated allele can be expressed in HGPS cells [128]. However, Reddel and Weiss (2004) demonstrated that both alleles, normal and mutated, can be found in HGPS cells due to partial abnormal splicing [129]. Progerin/LAΔ50 undergoes the same three enzymatic reactions as normal pre-lamin A, but the ZMPSTE24 cleavage site is lost as a result of truncation, thus preventing complete maturation. Progerin/LAΔ50 is anchored to the nuclear membrane and causes nuclear blebbing. It has been demonstrated that during mitosis, progerin/LAΔ50 is mislocalized into cytoplasmic aggregates and membranes, and can cause abnormal chromosome segregation as well as binucleation [120]. In addition, Buchwalter and Hetzer [130] showed that global protein synthesis, ribosome biogenesis and nucleoli activities are elevated in HGPS cells; they proposed that high nucleolar expression and rRNA production are hallmarks for premature aging, and limiting ribosome biogenesis could expand the lifespan. More recently, two humanized yeast systems were developed by Spear et al. [131] to examine the relation between the ZMPSTE24 enzyme and HGPS in the processing of pre-lamin A. These systems are powerful tools to study the C-terminal region of lamin A and to determine how it can affect the maturation process in normal and mutant HGPS cells.

NAT10 has also been linked to HGPS. In 2014, Larrieu et al. identified remodelin as a small chemical compound that targeted and inhibited NAT10, and which restored the nuclear shape of laminopathic cells and enhanced the organization of microtubules [7]. Chemical inhibition by remodelin was also tested on HGPS mice models, resulting in amelioration of health span, enhanced fitness and delayed appearance of HGPS phenotypes. Similar results were observed when one copy of *Nat10* was deleted in an HGPS mouse model: *Nat10+/*− HGPS mice were healthier than the aged wild type [8]. Using HGPS cells, Larrieu et al. were able to describe an important link between NAT10, RanGTP and Transportin-1 (TNPO1). Specifically, they reported that, in HGPS cells, the RanGTP gradient was defective, and Ran nuclear localization and abundance was decreased. This affected Ran’s import function and impaired its relationship with the non-classical transport pathway mediated by TNPO1, heterogenous nuclear ribonucleoprotein A1 (hnRNPA1) and NUP153. However, when NAT10 was inhibited, the proper nucleo-cytoplasmic ratio of TNPO1 was restored, and nuclear localization of hnRNPA1 and NUP153 was promoted, thereby allowing proper chromatin organization and transcription, as well as prevention of HGPS cells from senescence [9].

## 5. Concluding Remarks

**Figure 4:**
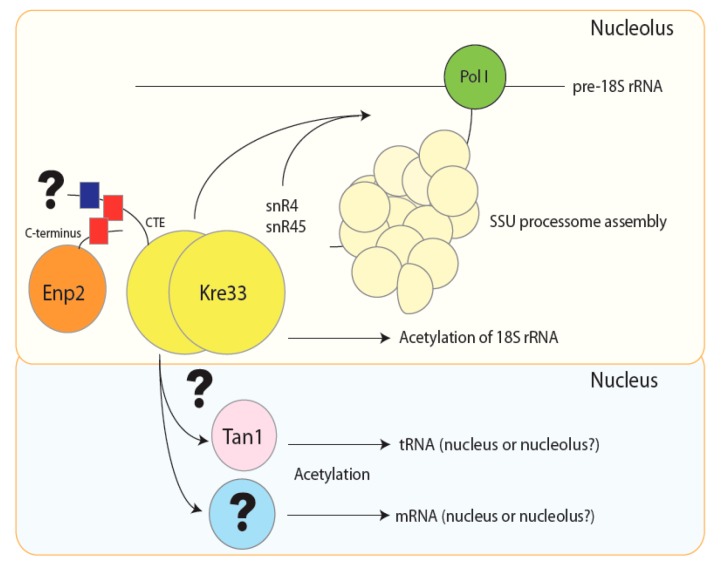
Hypothetical model for Kre33 acetylation of RNAs. In the nucleolus, the Kre33 homodimer and Enp2 protein may interact together via their C-terminal coiled-coil motifs (in red) before joining the SSU processome. In this particle, Kre33 catalyzes the acetylation of two 18S rRNA cytosines: each modification is guided by a different box C/D small nucleolar RNA (snoRNA), snR4 (C1280 in helix 34) and snR45 (C1773 in helix 45). Acetylation of tRNAs and mRNAs could occur in the nucleoplasm, where Kre33 needs to interacts with the adaptor protein Tan1 in order to acetylate tRNAs. It is not known if acetylation of mRNAs requires adaptor protein(s). Nevertheless, it remains possible that acetylation of tRNAs and mRNAs could take place in the nucleolus since tRNAs and mRNAs have previously been detected in this compartment [51,54,91,92].

In the past few years, cryo-EM structures of the *S. cerevisiae* SSU processome have been reported, revealing that Kre33 acetyltransferase forms a dimer in the SSU processome and connects Bms1 GTPase and Enp2 [66,68,73,132]. However, domains or sites of interactions between Kre33 and its partners are still not well defined. The coiled-coil motif in the CTE of Kre33 may be responsible for protein–protein interaction, but the current available structures of Kre33 in the SSU processome do not allow us to determine the position of the C-terminal extension, precluding the identification of the sites of interaction. In the SSU processome configuration, the active site of Kre33 is remote from its acetylation target sites in pre-18S rRNA. Following the release of many assembly factors and processing steps, pre-18S rRNA undergoes several conformational changes and pre-40S is more compact [66]. This raises questions about how and when Kre33 coordinates acetylation of pre-18S rRNA with SSU processome assembly and pre-18S rRNA processing. Acetylation of 18S rRNA is well conserved among eukaryotes, but the exact function of this modification remains elusive. Interestingly, Kre33 also acetylates tRNA^Ser^ and tRNA^Leu^, and creates a specific crosstalk between rRNAs and tRNAs [16]. Nevertheless, the regulation of this crosstalk in a spatiotemporal manner is still poorly understood. Does Kre33 need to interact with its adaptor protein Tan1 before or during the acetylation events? This remains an open question that will draw attention in the field (Figure 4).

Arango et al. [23] recently demonstrated that NAT10 acetylates mRNAs, thus enhancing their stability and translation efficiency. Their findings highlighted the role of NAT10 in the regulation of gene expression at the post-translational level. Future studies will undoubtedly reveal whether mRNA acetylation requires an adaptor protein as for tRNAs (Figure 4), if it is dynamic, and by which enzyme(s) this modification can be removed. NAT10 has been found to acetylate not only RNAs, but also proteins, such as histones and tubulins [7,112]. The inhibition of NAT10 restores the normal nuclear shape of HGPS patient cells due to microtubule deacetylation and cytoskeleton reorganization [7]. Eight sites of acetylation have been identified in lamin A and linked to chromatin tethering [133]. Yet, little is known about the link between NAT10 and acetylated lamin A. Despite the findings reported in this review article, many cellular functions of Kre33/NAT10 remain poorly understood and we foresee that deeper investigations combining biochemical techniques with structural analyses will lead to a better comprehension of this multifaceted enzyme.
